# An Integrative Pan-Cancer Analysis of PBK in Human Tumors

**DOI:** 10.3389/fmolb.2021.755911

**Published:** 2021-11-10

**Authors:** Huantao Wen, Zitao Chen, Min Li, Qiongzhen Huang, Yuhao Deng, Jiawei Zheng, Moliang Xiong, Pengfei Wang, Wangming Zhang

**Affiliations:** The National Key Clinical Specialty, The Engineering Technology Research Center of Education Ministry of China, Guangdong Provincial Key Laboratory on Brain Function Repair and Regeneration, Department of Neurosurgery, Zhujiang Hospital, Southern Medical University, Guangzhou, China

**Keywords:** pan-cancer, PBK, biomarker, tumor cell proliferation, prognosis

## Abstract

**Background:** PDZ binding kinase (PBK) is a serine/threonine kinase, which belongs to the mitogen-activated protein kinase kinase (MAPKK) family. It has been shown to be a critical gene in the regulation of mitosis and tumorigenesis, but the role of PBK in various cancers remains unclear. In this study, we systematically explored the prognostic and predictive value of PBK expression in 33 cancer types.

**Methods:** Public databases including the cBioPortal database, GDSC database, GTEx database, CCLE database, and TCGA database were used to detect the PBK expression and its association with the prognosis, clinicopathologic stage, TMB, MSI, immune microenvironment, immune checkpoints, immune cell infiltration, enrichment pathways, and IC50 across pan-cancer. The statistical analyses and visualization were conducted using R software.

**Results:** PBK expression is relatively high in most cancers compared to their normal counterparts, and this gene is barely expressed in normal tissues. High expression of PBK is significantly associated with poor prognosis and clinicopathologic stages I, II, and III in different cancers. Furthermore, PBK expression is strongly associated with TMB in 23 cancer types and associated with MSI in nine cancer types. Moreover, the correlation analysis of the microenvironment and immune cells indicated that PBK is negatively correlated with the immune infiltration levels but positively correlated with the infiltration levels of M0 and M1 macrophages, T cells CD4 memory activated, and T cells follicular helper. GSEA analysis revealed that the biological function or pathways relevant to the cell cycle and mitosis were frequently enriched at the level of high expression of PBK.

**Conclusion:** These results revealed the oncogenic role of PBK, which is significantly upregulated in various cancers and indicated poor prognosis and immune infiltration in multiple cancers. It also suggested that PBK may serve as a biomarker in multiple tumor progress and patient survival.

## Introduction

Cancer is the second most frequent cause of death worldwide and emerges in various forms according to cells of origin and the genomic alterations (Pan-cancer analysis of whole genomes 2020; Bray et al., 2013; Weinstein et al., 2013). The pan-cancer analysis project was launched by The Cancer Genome Atlas (TCGA), which aimed to find out the common features and the heterogeneities and analytical breadth across different cancers in 2012 (Weinstein et al., 2013; Liu et al., 2021). It is a systemic project to interpret molecular profiles from genomic, epigenomic, transcriptional, and proteomic levels in large cohorts. At the same time, calculation by repetitive timing and gene expression associated with background mutation rates enabled us to reduce false-positive and false-negative rates in statistics in a single type of tumor project (Lawrence et al., 2013; Weinstein et al., 2013). This helps us to comprehensively understand certain gene expression levels in cancer and the implication for cancer treatment in clinical practice.

PBK, also known as T-LAK cell-originated protein kinase (TOPK), is a serine/threonine kinase belonging to the MAPKK family, which is expressed exclusively in actively proliferating cells, particularly in germinal and fetal cells (Herbert et al., 2018). Compared to normal tissues, PBK has been proven to be upregulated in tumor tissues, such as breast cancer, lung cancer, and leukemia, which indicated a poorer cancer diagnosis and prognosis, making it a potential therapeutic target for cancer treatment (Park et al., 2006; Lei et al., 2013; Ishikawa, Senba, and Mori 2018; Han et al., 2021). PBK is also a crucial factor in proliferation, invasiveness, and metastasis in tumor tissues (Herbert et al., 2018; Hinzman et al., 2018; Q.X.; Yang et al., 2019). Recent study has suggested that PBK upregulates autophagy and chemoresistance through the ERK/mTOR signaling pathway in high-grade serous ovarian carcinoma (Ma et al., 2019). PBK overexpression also relates to the effect of anti-apoptosis in tumors from the previous confirmation studies (Park et al., 2013; Han et al., 2021). While most research to date has described the biological function of PBK in certain tumor types and limited samples, a comprehensive understanding of PBK in various cancers is needed.

In this study, we conducted a pan-cancer analysis to illustrate the biological functions of PBK across cancers. Different public databases were used to analyze the PBK expression in tumor and normal samples and the relationship with different cancers. In addition, the association between PBK and immune infiltration, mutation status, TMB, and MSI were explored across different cancers. Furthermore, co-expression of immune-related genes and PBK was conducted. Gene set enrichment analysis (GSEA) was used to explore the possible Gene Ontology (GO) function and pathway enriched in 33 cancer types. Finally, we performed the drug resistance analysis to figure out the correlation between PBK expression and half-maximal inhibitory concentration (IC50) in different antitumor drugs. This study provided novel insight into the functional role of PBK in different types of cancer and also revealed its potential correlation with immune infiltration and chemoresistance.

## Materials and Methods

### Data Processing and Expression Analysis

RNA-seq data, somatic mutation data, and relevant clinical data were downloaded from TCGA database, at the University of California Santa Cruz (UCSC, https://xenabrowser.net/). TOIL Genotype Tissue-Expression (GTEx) transcription expression RNA-seq and phenotype data were also obtained using UCSC Xena, and PBK expression was detected in 31 different tissues. The Cancer Cell Line Encyclopedia (CCLE, https://portals.broadinstitute.org/ccle/) database was used to collect each tumor cell line’s data, and 21 tissues were utilized to extract the PBK expression. Comparison of PBK expression between tumor and normal samples was conducted in 33 TCGA cancer types. All expression data were log2 transformed and the Wilcoxon test was used to compare the difference significance between tumor and normal samples. In addition, PBK expression was also detected in 21 tumor cell lines and 31 normal tissues; Kolmogorov–Smirnov tests were conducted after the expression data were log2 transformed.

### Survival analysis of PBK and its correlation with clinical phenotypes in different cancers

Survival analysis was then processed using Kaplan–Meier methods and the log-rank test. We selected four indicators to explore the relationship between PBK expression and patient prognosis, including overall survival (OS), disease-specific survival (DSS), disease-free interval (DFI), and progression-free interval (PFI). The Kaplan–Meier curve was plotted using R packages “survival” and “survminer.” Meanwhile, Cox analysis was processed and visualized using R packages “survival” and “forestplot.” We also detected the PBK expression and their association with the tumor stage in different cancer types, which was performed using R packages “limma” and “ggpubr.”

### Mutation Profiles

The cBio Cancer Genomics Portal (cBioPortal, http://cbioportal.org) is an open platform for multidimensional cancer genomic research (Cerami et al., 2012). 32 “TCGA Pan Cancer Atlas Studies” and 10967 samples were chosen to investigate the copy number alteration (CNA), mutation rates, and distribution of PBK. The catalog of somatic mutations in cancer (COSMIC, https://cancer.sanger.ac.uk/cosmic/) covers all resources for the effect and the genetic mechanism of somatic mutations in human cancer (Tate et al., 2019; Ye et al., 2021). The COSMIC database was used to explore the mutation of PBK in specific cancer types in this study.

### Correlation of PBK Expression With Tumor Mutation Burden, Tumor Microsatellite Instability, and Immune Checkpoint Expression

TMB represents all mutation numbers in a tumor tissue, which serve as a biomarker for the sensitivity of immune checkpoint inhibitor therapy in several cancers (Choucair et al., 2020). We calculated TMB scores using the mutation data downloaded above and determined the correlation between PBK expression and TMB scores. MSI means the genetic hypermutability caused by impaired DNA mismatch repair, which may also influence the immune checkpoint therapy (Yamamoto and Imai 2019). MSI scores were also calculated, as well as their association with PBK expression. The results are visualized as a radar chart using the R package “fmsb.” Furthermore, we evaluated the association between PBK expression and the immune checkpoint previously reported (Paluch et al., 2018; Pan et al., 2019; Liu et al., 2021). The result is presented as a heatmap which was generated using the “reshape2” and “RColorBrewer” packages.

### PBK Expression and Immune Infiltration

Immune scores, stromal scores, estimate scores, and tumor purity were calculated using the ESTIMATE algorithm based on transcriptome expression profiles in various cancers, which are frequently used to assess the degree of infiltration of stromal or immune cells (Yoshihara et al., 2013). Then, the correlations of PBK expression with immune and stromal scores in different cancers were presented as scatter plots. Additionally, we calculated the proportion of 22 tumor-infiltration immune cells in each pan-cancer patient based on the CIBERSORT algorithm, which can predict the composition of immunocytes based on the gene expression profiles of tumor tissues (Newman et al., 2015). Moreover, the association between PBK expression and immune cells was assessed and visualized using the packages “ggplot2,” “ggpubr,” and “ggExtra.”

### Gene Set Enrichment Analysis

GSEA analysis was performed to identify the biological function of PBK in different tumors. We downloaded the gene sets “c5.go.v7.3.symbols” and “c2.cp.kegg.v7.3.symbols” from the official GSEA website (https://www.gsea-msigdb.org/gsea/downloads.jsp), which represents the GO and Kyoto Encyclopedia of Genes and Genomes (KEGG) gene sets, respectively. We consider *p*<0.05 as the significance threshold, and R packages “limma,” “org.Hs.eg.db,” “clusterProfiler,” and “enrichplot” were used to plot enrichment results.

### Drug Sensitivity Analysis

The response to chemotherapy for each sample was predicted based on the Genomics of Drug Sensitivity in Cancer (GDSC, https://www.cancerrxgene.org/) (W. Yang et al., 2013). Cell line data, IC50 information of different compounds, and the gene expression profile were downloaded from the GDSC database. We extracted the PBK expression and IC50 value using strawberry Perl (version 5.30.1, http://strawberryperl.com/), and then the correlation between PBK expression and each compound’s IC50 value was shown as scatter plots using the R packages “ggplot2,” “ggpubr,” and “ggExtra.”

### Statistical Analysis

All statistical analyses were conducted using R software (version 4.0.2) and attached packages. The Wilcoxon test was used to compare the gene expression between the normal and tumor samples in all the tumors across all cancer types. The Kruskal–Wallis test was used to compare the difference in gene expression between different tumor stages and the gene expression in different tumor cell lines and normal tissues. Survival analyses were performed using the log-rank test, the Kaplan–Meier method, and the Cox regression model. Correlation analyses were conducted using Pearson’s test. Two-tailed *p* value <0.05 was considered as statistically significant.

## Results

### PBK Expression in Tumor and Normal Tissue Samples

**FIGURE 1:**
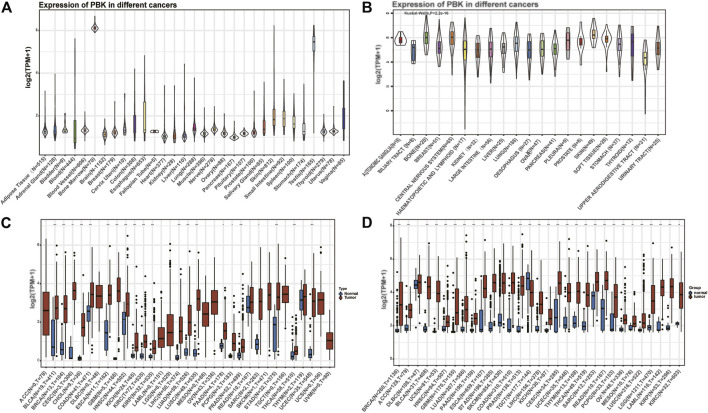
**|** PBK is abnormally expressed in pan-cancer. **(A)** PBK expression in 31 tissues from the GTEx database. **(B)** PBK expression in 21 tumor cells from the CCLE database. **(C)** Differential expression of PBK in cancers and normal tissues from TCGA database. **(D)** PBK is abnormally overexpressed in 30 cancer types from the GTEx database and TCGA database (*p < 0.05, **p < 0.01, ***p < 0.001).

We first analyzed the expression pattern of PBK in nontumor tissues and different tumor cell lines. As shown in Figure 1A, PBK is highly expressed in the testis and bone marrow tissues, while it is lowly expressed in brain, breast, liver, and other normal tissues. Next, the expression of PBK in 21 tumor cell lines based on the CCLE database showed that PBK displayed inconsistent expression levels across tumor cell lines (*p* = 2.2e-16, Figure 1B), and the expression of the PBK level was relatively high in tumor cells. We next compared the difference of PBK mRNA expression between cancer and normal tissues on TCGA database, and the results showed that PBK was relatively highly expressed in most tumor tissues (*p* < 0.05), except for SKCM, THYM, and READ (Figure 1C). Considering that the amount of the normal samples is relatively small in TCGA database, we combined the TCGA database and the GTEx database to analyze the difference of PBK expression levels in 31 cancer types (Figure 1D). We also found that PBK was significantly upregulated in cancer samples compared to their paired normal samples (*p* < 0.01), consistent with the results from TCGA, except for DLBC and TGCT.

These results above indicated that PBK is aberrantly overexpressed in most tumor types.

### Correlation of PBK and Clinical Features

**FIGURE 2:**
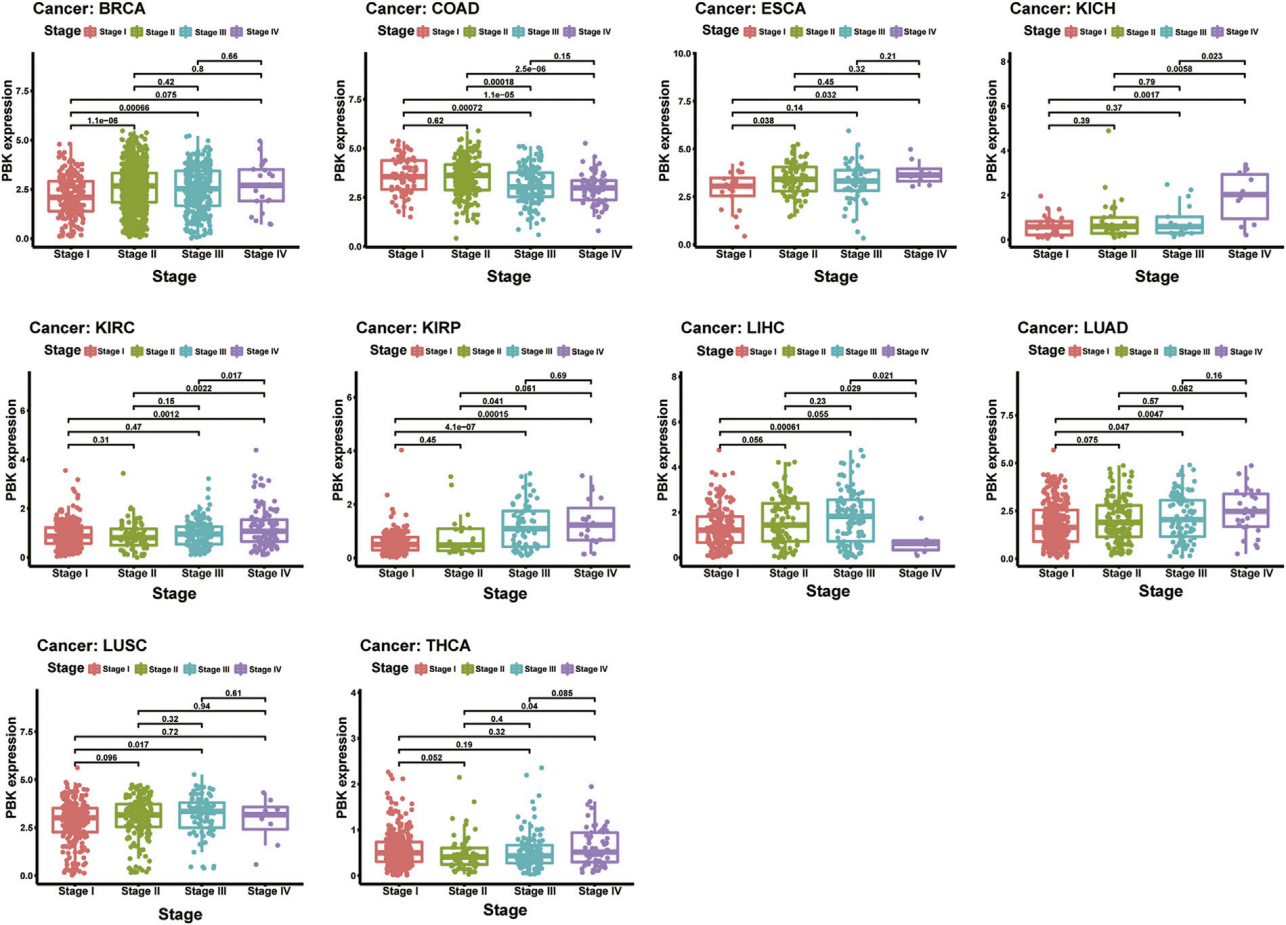
**|** Relationship of PBK expression with patients’ OS. Forest plots of hazard ratios of PBK in 33 cancer types and Kaplan–Meier OS curves for patients stratified by different expression levels of PBK in eight cancer types.

We next investigated the PBK expression in different tumor stages across cancers and compared the expression in different stages in each tumor type. As shown in Figure 2, PBK is upregulated in higher tumor stages in most cancers including BRCA, ESCA, KICH, KIRC, KIRP, and LUAD. It is upregulated in stages I, II, and III and downregulated in stage IV in COAD and LUSC. Also, PBK is downregulated in COAD during increased tumor stages. PBK expression was significant in stages I, II, and III across cancers, and the differences between higher tumor stages were relatively small and not statistically significant.

The results above showed that PBK expression is positively associated with the stage of the cancer in most tumor types.

### Expression of PBK Is Associated With Patient Prognosis

**FIGURE 3:**
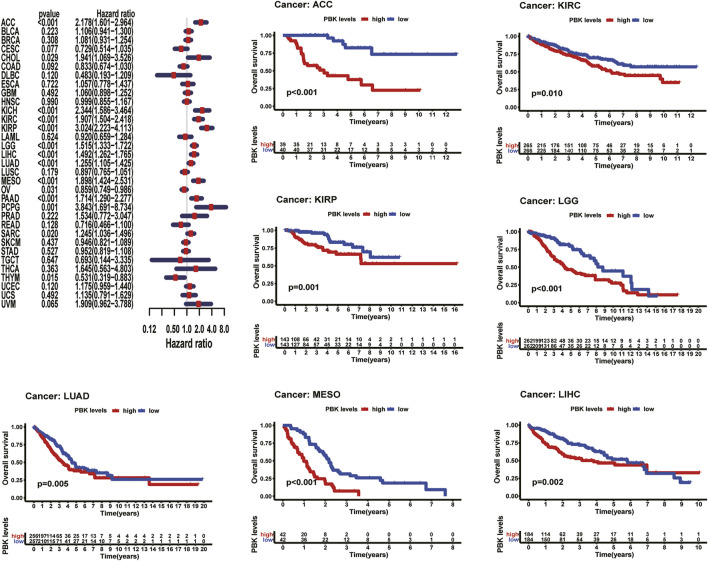
**|** Relationship of PBK expression with patients’ DSS. Forest plots of hazard ratios of PBK in 33 cancer types. Kaplan–Meier PFI curves for patients stratified by different expression levels of PBK in seven cancer types.

**FIGURE 4:**
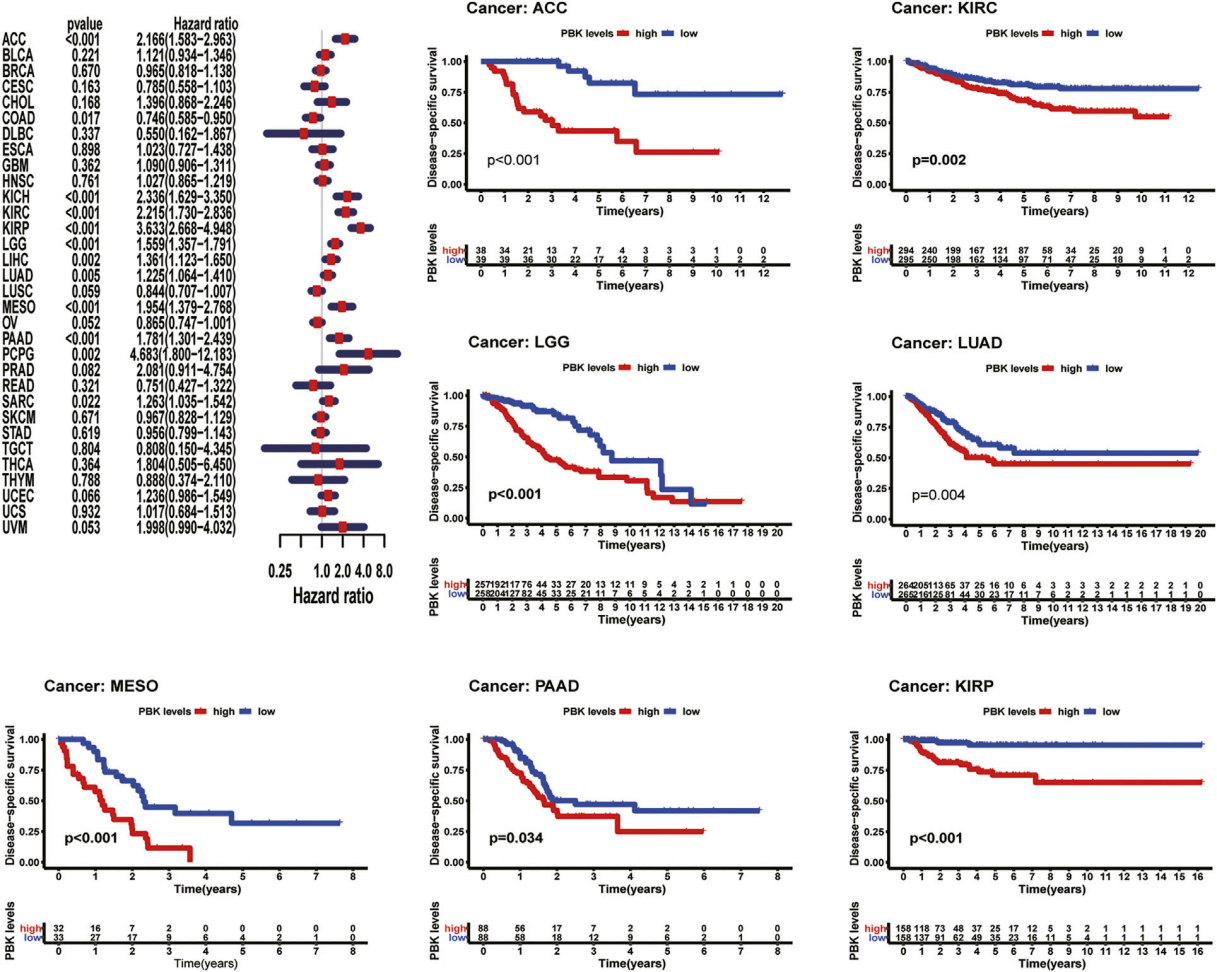
**|** Relationship of PBK expression with patients’ PFI. Forest plots of hazard ratios of PBK in 33 cancer types. Kaplan–Meier PFI curves for patients stratified by different expression levels of PBK in nine cancer types.

As shown in Figure 3, Kaplan–Meier plots identified that high expression of PBK indicated poorer prognosis in ACC (*p* < 0.001), KIRC (*p* = 0.01), KIRP (*p* = 0.001), LGG (*p* < 0.001), LIHC (*p* = 0.002), MESO (*p* < 0.001), and LUAD (*p* = 0.005), and Cox regression analyses confirmed that PBK is a high-risk gene in these cancer types, as well as in CHOL (HR = 1.941, *p* = 0.029), KICH (HR = 2.344, *p* < 0.001), DAAD (HR = 1.714, *p* < 0.001), and PCPG (HR = 3.843, *p* = 0.001) (Figure 3). By univariate Cox analysis in DSS of 33 cancer types, we found that high PBK expression is associated with poorer prognosis in ACC (HR = 2.166, *p* < 0.001), KICH (HR = 2.336, *p* < 0.001), KIRC (HR = 2.215, *p* < 0.001), KIRP (HR = 2.867, *p* < 0.001), LGG (HR = 1.319, *p* < 0.001), LIHC (HR = 3.633, *p* < 0.001), LUAD (HR = 1.225, *p* < 0.001), MESO (HR = 1.954, *p* < 0.001), PCPG (HR = 4.683, *p* = 0.002), and SARC (HR = 1.263, *p* = 0.022) (Figure 4). Kaplan–Meier curves showed that an increased PBK expression correlated with poor prognosis in ACC (*p* < 0.001), KIRC (*p* = 0.002), LGG (*p* < 0.001), LUAD (*p* = 0.004), MESO (*p* < 0.001), PAAD (*p* = 0.034), and KIRP (*p* < 0.001) (Figure 4).

**FIGURE 5:**
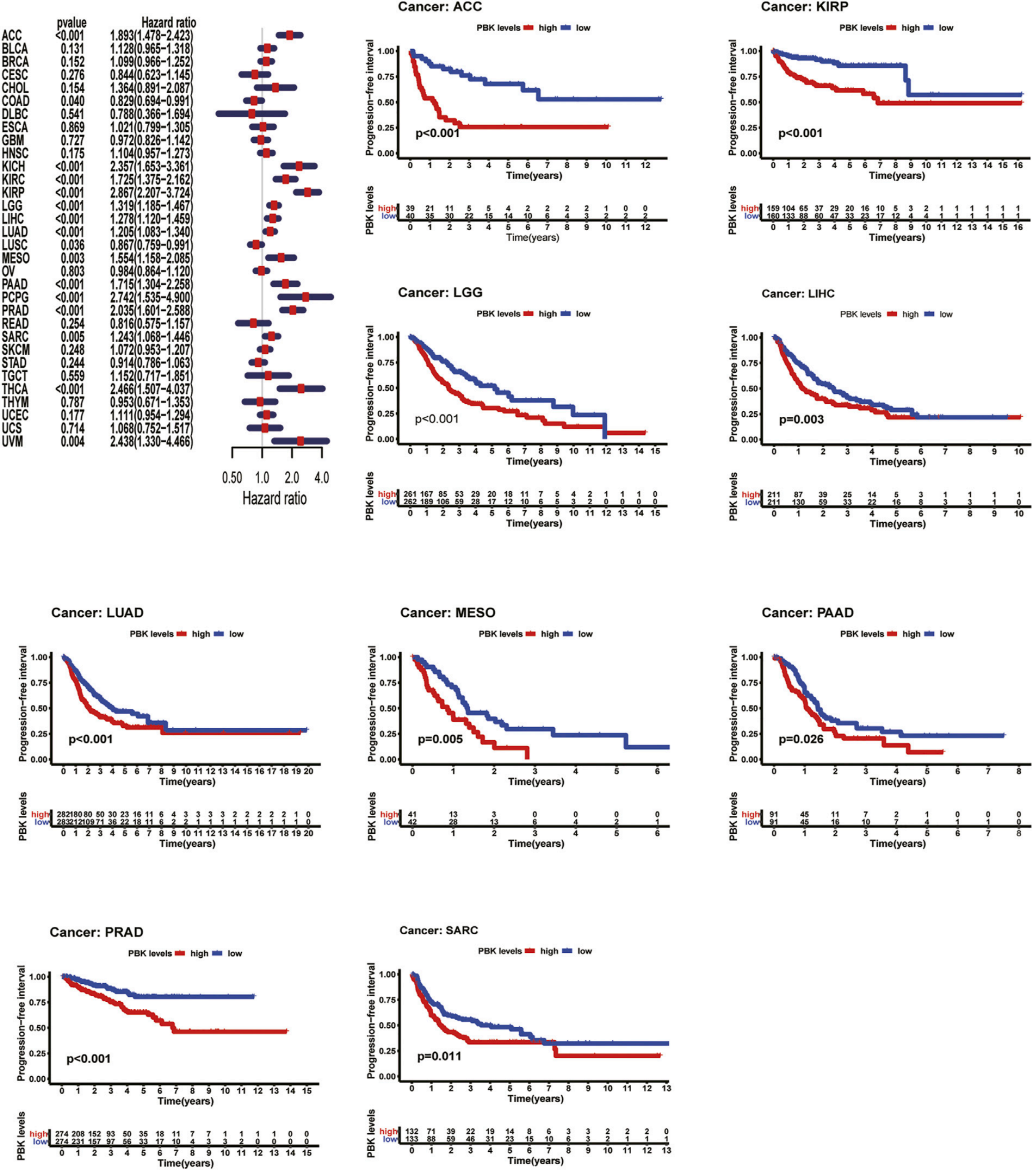
**|** Relationship of PBK expression with patients’ DFI. Forest plots of hazard ratios of PBK in 33 cancer types. Kaplan–Meier PFI curves for patients stratified by different expression levels of PBK in five cancer types.

Cox hazards model analysis in PFI indicated that PBK is a high-risk factor in ACC (HR = 1.893, *p* < 0.001), KICH (HR = 2.357, *p* < 0.001), KIRC (HR = 1.725, *p* < 0.001), KIRP (HR = 2.867, *p* < 0.001), LGG (HR = 1.319, *p* < 0.001), LIHC (HR = 1.278, *p* < 0.001), LUAD (HR = 1.278, *p* < 0.001), MESO (HR = 1.554, *p* = 0.003), PAAD (HR = 1.715, *p* < 0.001), PCPG (HR = 2.742, *p* < 0.001), PRAD (HR = 2.035, *p* < 0.001), SARC (HR = 1.243, *p* = 0.005), THCA (HR = 2.466, *p* < 0.001), and UVM (HR = 2.438, *p* = 0.004) (Figure 5). Moreover, Kaplan–Meier methods revealed that PBK indicated worse PFI in ACC (*p* < 0.001), KIRP (*p* < 0.001), LGG (*p* < 0.001), LIHC (*p* = 0.003), LUAD (*p* < 0.001), MESO (*p* = 0.005), PAAD (*p* = 0.026), PRAD (*p* < 0.001), and SARC (*p* = 0.011) (Figure 5).

**FIGURE 6:**
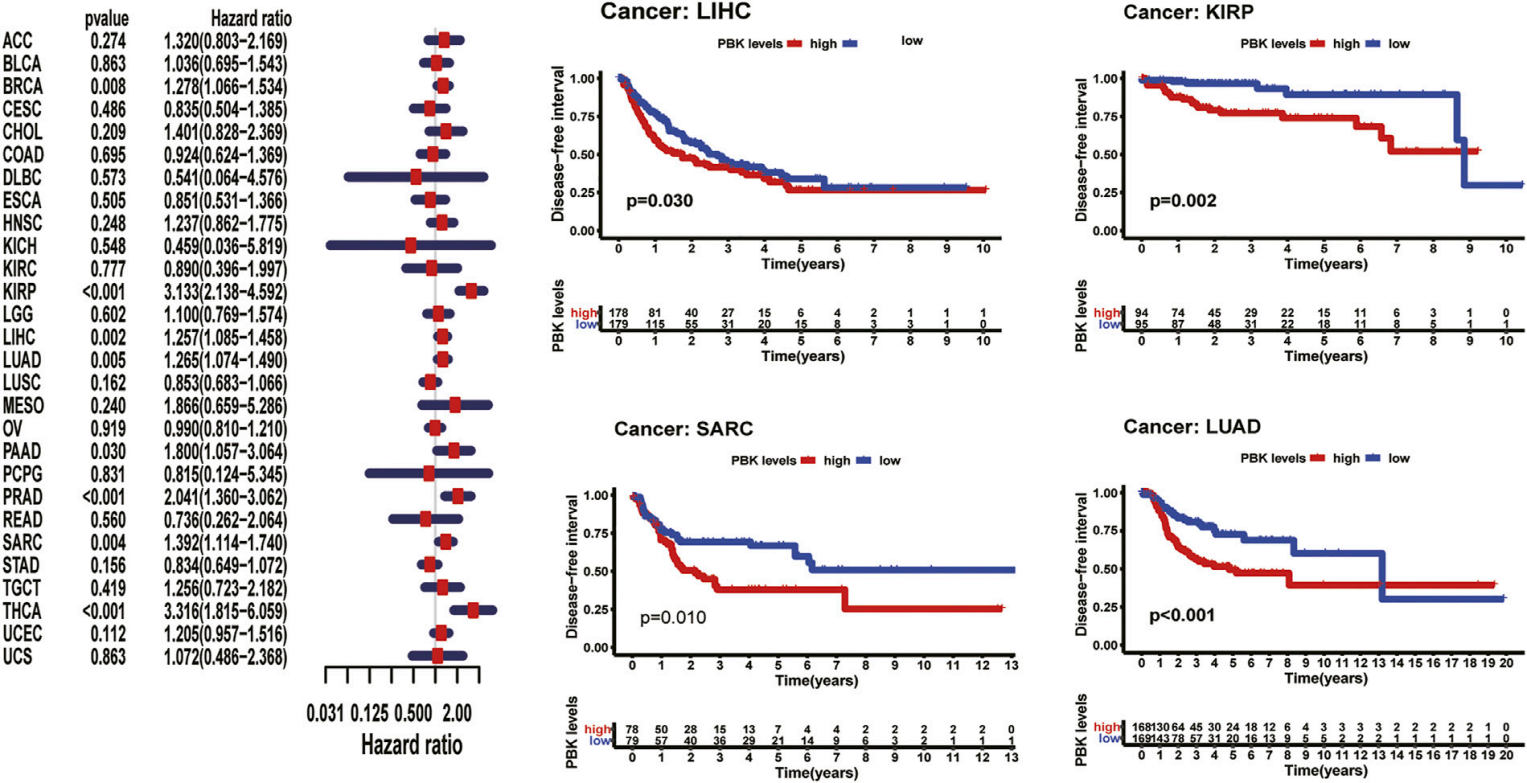
**|** Relationship between PBK expression and the clinicopathologic stage in 10 tumors.

Meanwhile, we also analyzed DFI and revealed that high PBK expression indicated a risk in BRCA (HR = 1.278, *p* = 0.008), KIRP (HR = 3.133, *p* < 0.001), LIHC (HR = 1.257, *p* = 0.002), LUAD (HR = 1.265 *p* = 0.005), PAAD (HR = 1.800, *p* < 0.03), PRAD (HR = 2.041, *p* < 0.001), SARC (HR = 1.243, *p* = 0.004), and THCA (HR = 3.316, *p* < 0.001) (Figure 6). Kaplan–Meier DFI curves indicated that patients with high PBK expression showed inferior prognosis in KIRP (*p* = 0.002), LIHC (*p* = 0.030), LUAD (*p* < 0.001), and SARC (*p* = 0.010) (Figure 6).

The results above showed a negative correlation between PBK expression and patient survival time in most types of cancer. Kaplan–Meier analysis also suggests a poorer prognosis in these cancers, including ACC, LUAD, LIHC in OS, PFI, DSS, or DFI.

### PBK Mutations in Different Tissues

**FIGURE 7:**
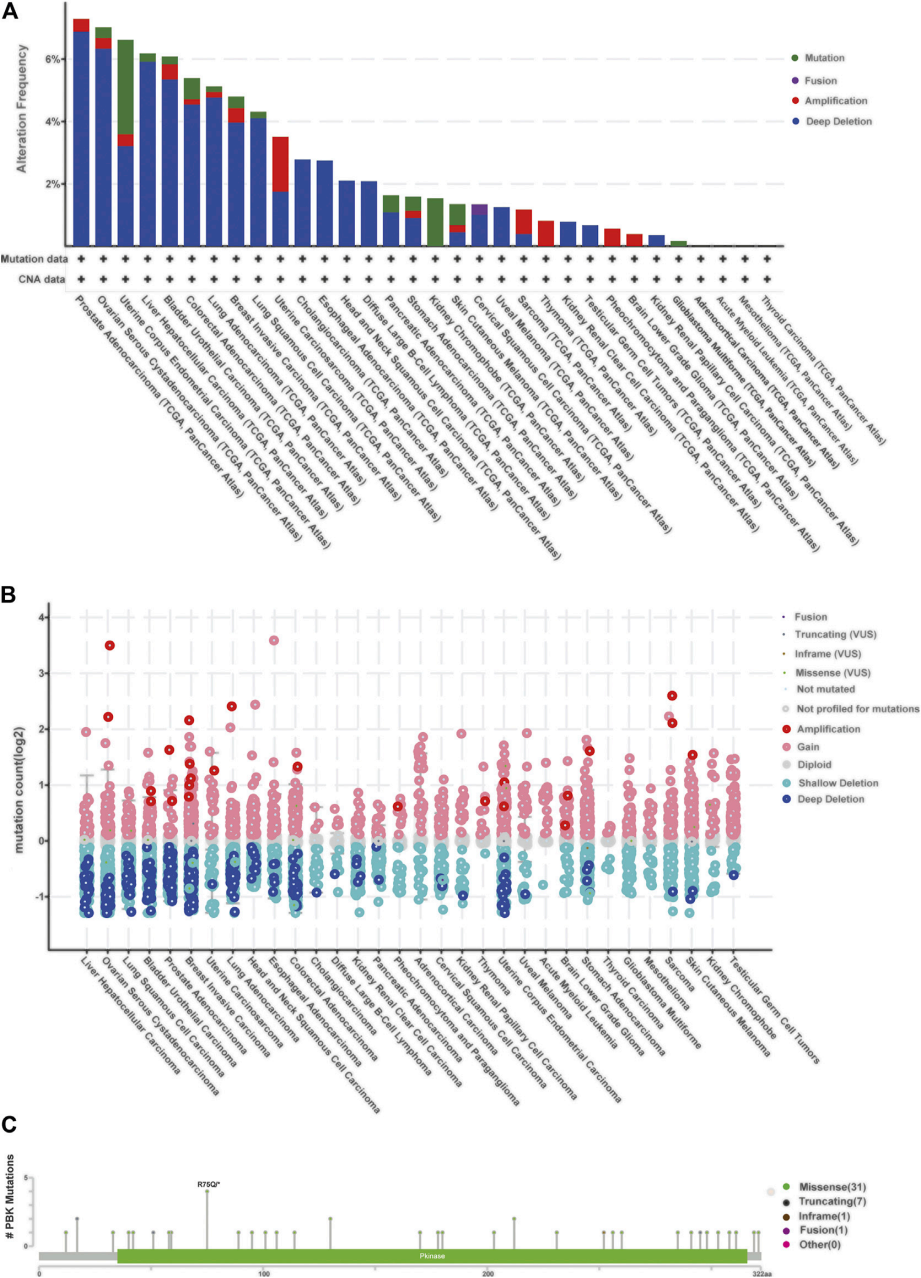
**|** Relationship between PBK and mutation in human cancers based on the cBioPortal database. **(A)** PBK mutation level in pan-cancer; **(B)** mutation count of PBK in different cancer types; **(C)** mutation diagram of PBK in different cancer types across protein domains.

We then observed the alteration frequency and status based on TCGA pan-cancer cohorts in cBioportal. We found that prostate tumors, ovarian cancer, uterine tumors, liver cancer, and bladder cancer shared high alteration frequency (>6%) with “deep deletion” in major mutation, as well as in most cancer types in these cohorts (Figure 7A). Also, the “Amplification” type serves as the major CNA type in sarcoma, thymoma, low grade glioma, and pheochromocytoma and paraganglioma, as shown in Figures 7A, B. There are 40 mutation sites detected in the location between amino acids 0 and 322, including 31 missense, seven truncating, one inframe, and one fusion (Figure 7C), and R75Q is the most frequent mutation site.

The Cosmic database provided detailed information about mutation types that correlate with PBK in different cancers, which contains nonsense substitution, missense substitution, synonymous substitution, frameshift substitution, inframe deletion, and others (Supplementary Figure S1). Nonsense substitutions were found in bone cancer (50%), breast cancer (12.50%), large intestine cancer (2.63%), liver cancer (4%), and skin cancer (12.50%). Missense substitution exists in breast cancer (29.17%), central nervous system (CNS) cancer (50%), endometrium cancer (58.33%), haematopoietic and lymphoid cancer (30.77%), kidney cancer (25%), large intestine cancer (34.21%), liver cancer (20%), lung cancer (64.29%), ovary cancer (28.57%), pancreas cancer (6.67%), skin cancer (50%), soft tissue cancer (50%), stomach cancer (22.73%), thyroid cancer (100%), and urinary tract cancer (100%). The proportion of synonymous substitution is 8.33% in breast cancer and endometrium cancer, 18.42% in large intestine cancer, 14.29% in lung cancer, 13.33% in prostate cancer, 50% in soft tissue cancer, 18.18% in stomach cancer, and 50% in upper aerodigestive cancer. Also, C > T and G > A types were predominantly observed in the PBK coding strand mutations (Supplementary Figure S1). Other mutation types like frameshift substitution and inframe deletion seldom occurred in different cancers.

In summary, the common PBK mutation types in most cancers are nonsense substitution, missense substitution, and synonymous substitution.

### Correlation Analysis With TMB, MSI, Tumor Microenvironment, and Immune Markers

**FIGURE 8:**
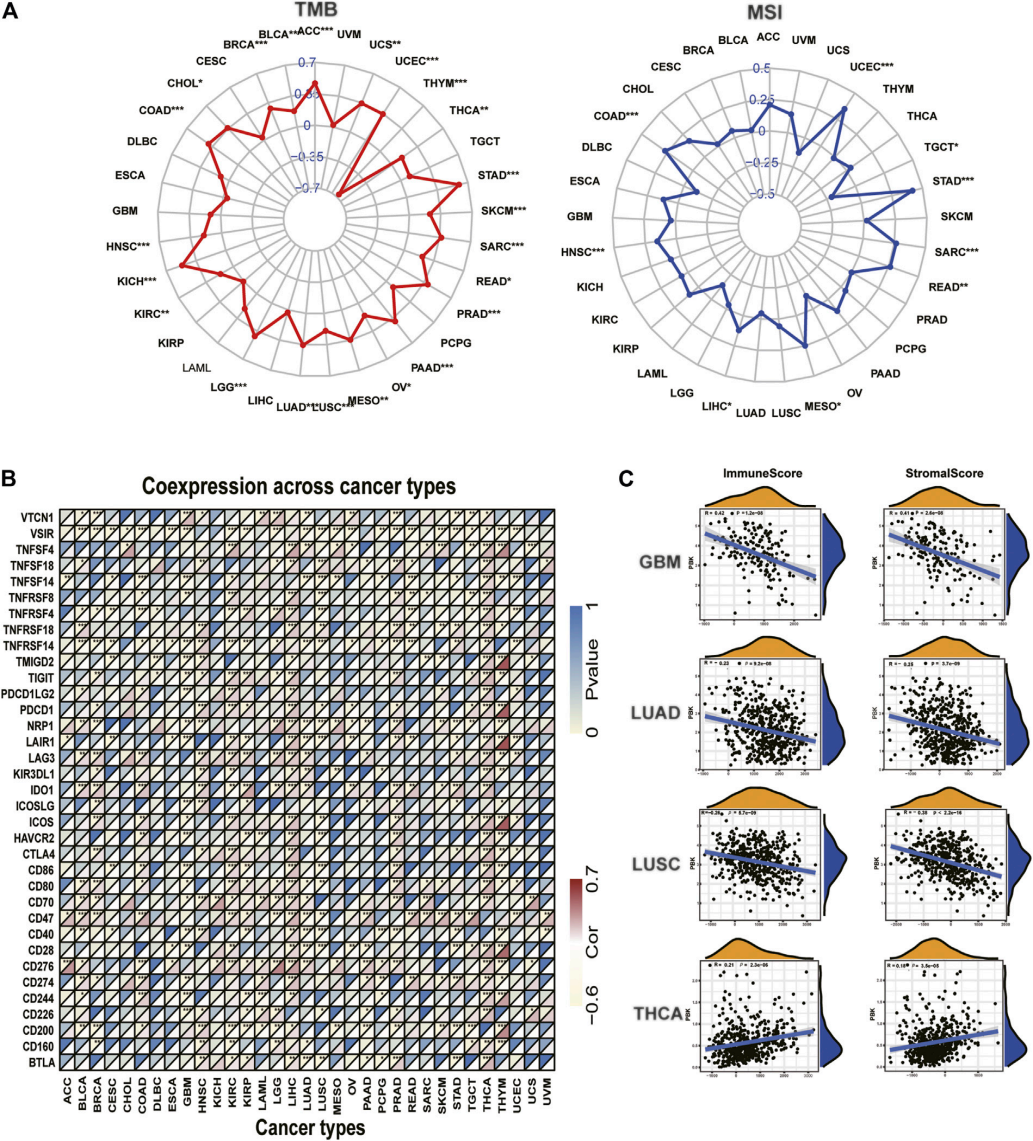
**|** Correlation of PBK expression and the TMB, MSI, and tumor immune microenvironment. **(A)** Radar graphs of correlation of PBK with TMB. **(B)** Radar graphs of correlation of PBK with MSI. The coordinate value corresponding to each point is the correlation coefficient. (***p < 0.001; **p < 0.01; *p < 0.05). **(C)** Correlation of PBK with the immune score and the stromal score.

According to the estimate algorithm, we calculated the immune, stromal, and estimate scores and tumor purity in different cancers. Then, we analyzed PBK expression and the correlation with the stromal score and the immune score. The results showed that PBK is negatively correlated with the stromal score and the immune score in most cancer types, apart from THCA. The most significant four tumors in correlation analysis were presented in Figure 8D, including GBM, LUAD, LUSC, and THCA. Results in other cancers are presented in Supplementary Table S1. Considering the significant characteristic with the sensitivity of immune checkpoint inhibitors, we next explore the correlations with PBK expression and TMB and MSI. We analyzed the correlation between the PBK expression level and TMB, which showed positive correlation with TMB in 22 types of cancer, including STAD, UCEC, COAD, SARC, READ, MESO, and HNSC (Figure 8A), while it is negatively correlated with THYM, which is the only tumor type with negative correlation. Notably, PBK expression is positively relevant to MSI in STAD, UCEC, COAD, SARC, READ, MESO, HNSC, and LIHC (Figure 8B) but negatively correlated with MSI in TGCT.

Furthermore, we investigated the relationships between PBK expression and immune checkpoints markers in 33 tumors. More than 40 immune checkpoints were selected, and the correlation heatmap was displayed in Figure 8C. Our results showed that PBK is significantly correlated with the expression of some immune markers, such as V-Set Immunoregulatory Receptor (VSIR), Neuropilin 1 (NRP1), TNF Receptor Superfamily Member 14 (TNFRSF14), Leukocyte Associated Immunoglobulin Like Receptor 1 (LAIR1), CD47, CD276, and CD200. We also found that PBK is positively correlated with the immune checkpoint expression in LIHC, THCA, and KIRC. This suggests that PBK might be relevant to tumor immunity by regulating the expression levels of these immune checkpoints. The comprehensive information of all 33 types of cancer is shown in Supplementary Table S2.

These results above showed that PBK is negatively correlated with the immune and stromal scores in most cancer types and positively correlated with the immune checkpoint expression in some cancers.

### Association Between PBK Level and Immune Cell Infiltration

**FIGURE 9:**
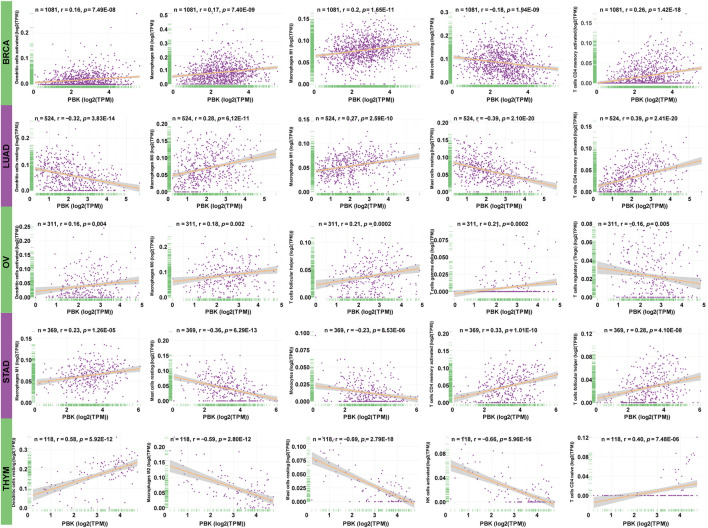
**|** Association between PBK expression and tumor infiltration of different immune cells in five tumor types.

To clarify the association between PBK expression and specific immune cell types in pan-cancer, we calculated the immunocyte compositions of all TCGA patients using the CIBERSORT algorithm and then explored their correlation with different PBK expression levels. The five most significant cancer types are presented in Figure 9, with the five most relevant immune cells. The detailed information of other tumors is included in Supplementary Table S3. We found that PBK is negatively associated with dendritic cells resting, monocytes, T cells regulatory (Tregs), and mast cells resting, except for THYM. But it is positively correlated with macrophages M0 (except for THYM), T cells CD4 memory activated (except for THYM), NK cells activated (except for THYM), and T cells follicular helper in these five tumors.

The study about immune cell infiltration revealed that PBK is negatively related to immune infiltration.

### Gene Set Enrichment Analysis in Different Cancers

**FIGURE 10:**
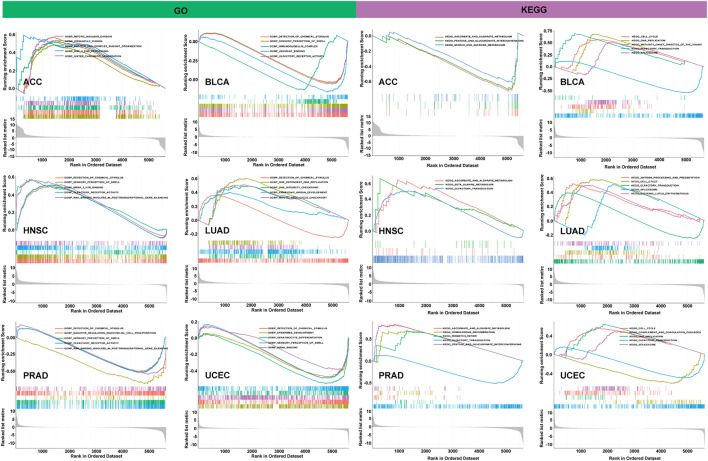
**|** GO term and KEGG pathway enrichment plots from GSEA analysis.

To observe the biological effects of PBK expression in pan-cancer, all TCGA samples were divided into PBK high-expression and low-expression groups, according to the PBK median expression level, and then we performed the GSEA analysis in GO and KEGG pathway analysis, and the results of six tumors are shown in the left of Figure 10. In GO terms, PBK is negatively correlated with detection of chemical stimulus in BLCA, PRAD, and UCEC but positively regulated with the detection of chemical stimulus in HNSC and LUAD. We also found that PBK is positively associated with mitotic nuclear division and sister chromatid segregation in ACC, as well as DNA-dependent DNA replication, the DNA integrity checkpoint, and the mitotic cell cycle checkpoint in LUAD. In KEGG terms, the PBK high-expression level is positively involved in the cell cycle in BLCA, LUAD, and UCEC and also positively correlated with DNA replication in BLCA and UCEC. Olfactory transduction also showed significant enrichment in BLCA, HNSC, PRAD, and UCEC (Figure 10).

Our results indicate that PBK is widely involved in the regulation of the function and relevant signaling pathways involved in the cell cycle and DNA replication.

### The Sensitivity to Chemotherapy and Target Therapy With PBK Expression

**FIGURE 11:**
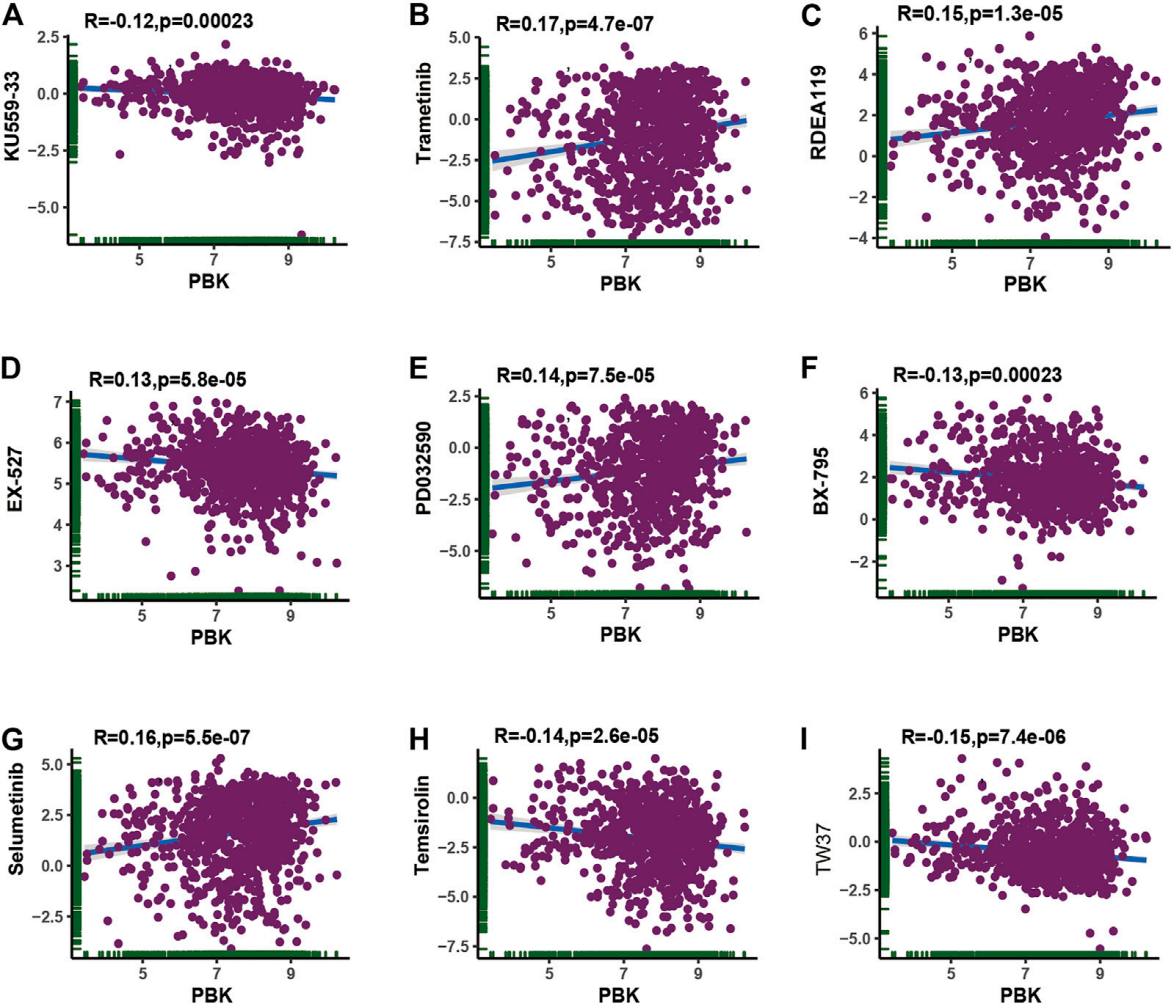
**|** Correlation of sensitivity to chemotherapy (IC50) with PBK expression.

Considering that chemotherapy and targeted therapy is the common way in cancer therapy and previous study indicated that PBK might promote chemotherapy resistance (Zykova et al., 2006; Zykova et al., 2010; Ma et al., 2019), we tried to assess the association between PBK expression and the IC50 level from each GDSC cell line dataset. The top nine absolute values of the highest levels of correlation about the PBK expression and IC50 value of different compounds in pan-cancer cell lines are shown in Figures 11A–I. Our results showed that PBK expression is positively correlated with trametinib, RDEA119, PD032590, and selumetinib, while it is negatively correlated with KU559-33, EX-527, BX-795, temsirolin, and TW37. The pan-cancer IC50 analysis results are available in Supplementary Table S4.

Conclusively, the PBK expression level was positively correlated with trametinib, RDEA119, PD032590, and selumetinib, which indicated that PBK may be positively correlated with the treatment of these chemotherapeutic drugs and may provide a new direction in tumor chemotherapy.

## Discussion

PBK, formed by 322 amino acids, is identified as a protein kinase which belongs to the MAPKK family (Gaudet, Branton, and Lue 2000; Dong et al., 2016). Its expression and phosphorylation levels continued to increase when proliferating cells entered mitosis (Fujibuchi et al., 2005). PBK expression is closely related to the inactivation of protein phosphatase 1α (PP1α); this process was regulated by the CDK1/cyclin B complex (Abe et al., 2007; Park et al., 2010). It is expressed exclusively in germinal and fetal cells and barely expressed in other normal tissues (Abe et al., 2007), while it is overexpressed in breast cancer, gastric cancer, esophageal cancer, glioblastoma, ovarian cancer, and hematologic tumors (Simons-Evelyn et al., 2001; Park et al., 2006; Dou et al., 2015; Ohashi et al., 2016; Ohashi et al., 2017; Kruthika et al., 2019; Ma et al., 2019). In our study detecting the expression of PBK in pan-cancer, we found that PBK is highly expressed in various types of cancers, including ACC, LGG, LUAD, and OV, as well as in tumor cell lines, while PBK is barely expressed in normal tissues, except for bone marrow and the testis, which is consistent with previous reports that PBK is abnormally expressed in tumor tissues and proliferating cells but is hardly detected in normal tissues. The results first showed that the expression level of PBK was relatively lower in TGCT than in its paired normal tissues as there is no previous report on PBK functions in TGCT. But the further mechanism has not been studied. Subsequently, we explored whether PBK expression is correlated with patient prognosis, and the results showed a negative correlation between PBK expression and patient survival time in most types of cancer. Kaplan–Meier analysis also suggests a poorer prognosis in these cancers, including ACC, LUAD, LIHC in OS, PFI, DSS, or DFI. Lei et al. (2015) reported that PBK/TOPK positively correlates with mutant p53 and affects cell proliferation and viability in lung adenocarcinoma, also associated with poor prognosis and an advanced tumor stage. Recent study also suggested that PBK is significantly upregulated, and PBK promotes invasion and migration *via* the ETV4-uPAR signaling pathway in hepatocellular carcinoma (Q.X. Yang et al., 2019). In contrast, high expression of PBK serves as a low-risk biomarker of OV in OS and DSS analyses. Recent study suggested that high PBK expression was correlated with a poor prognosis, metastasis, and cisplatin resistance in high-grade serous ovarian carcinoma (HGSOC) (Ma et al., 2019); this indicated that the role of PBK in ovarian cancers still needs more investigation. Meanwhile, we also found that PBK expression was correlated with the tumor stage in various cancers. Especially between stage I, II, and III tumors, PBK expression is significantly different. Interestingly, we found that the PBK expression in COAD and LUSC is downregulated in the advanced stage, and then we also investigated the association of PBK expression and COAD and LUSC prognosis, but the result showed that there are no significant differences between high- and low-PBK expression groups. Does that mean COAD and LUSC are different to other cancers? Further investigation is needed. Our study demonstrated that PBK may serve as a biomarker to determine the early stages in various cancers.

Genetic alteration might lead to the malignancy and proliferation in tumor tissues. The work of Ohashi et al. (2017) showed that PBK knockdown inhibits gastric carcinoma cell proliferation through p53 activation in a TP53 mutation-dependent manner. There is no previous study recognizing the mutation types and numbers of PBK. In our study, we revealed that PBK is mainly involved in nonsense substitutions and missense substitutions. Immunotherapy has achieved great success in cancer therapy, and multiple biomarkers have been reported to predict the response to immunotherapy, as well as TMB and MSI (Doroshow et al., 2019). However, immunotherapy still remains ineffective in many patients (Hegde and Chen 2020). The tumor microenvironment and the interactions between peripheral immune cells and tumor cells have a great influence on immunotherapy responses (Hiam-Galvez, Allen, and Spitzer 2021). The role of PBK in the tumor immune landscape is still unclear. Previous study indicated that PBK/TOPK might be an immunotherapy target in bladder cancer (Singh et al., 2014). We first examined the TMB and MSI scores in pan-cancer and their relationships with PBK expression levels, based on the TCGA data. Our results showed that PBK is positively correlated with TMB in 22 cancer types and is significantly correlated with THYM in TMB. Moreover, PBK expression is positively relevant to MSI in nine cancer types, most of which also positively correlated with TMB. The research of Chalmers et al. (2017) indicated that the majority of high-MSI patients also possess high TMB scores. Our results indicated that PBK expression may affect the TMB and MSI levels in different cancers and their immunotherapy responses. In the correlation analysis of PBK and immune checkpoints, PBK is significantly correlated with VSIR, NRP1, TNFRSF14, LAIR1, CD47, CD200, and CD276. Consistent with recent study, Wang et al. (2021) suggested that PBK promotes the CD276 transcription through the enrichment of the MSL complex, which plays an important role in the immune evasion of nasopharyngeal carcinoma (NPC), and may serve as a biomarker for cancer immunotherapy. This suggests that PBK expression might be relevant to the immunotherapy in these immune checkpoints.

Next, we carried on the microenvironment of pan-cancer based on the ESTIMATE method, obtaining the immune and stromal scores in pan-cancer. Further analysis showed that PBK is negatively correlated with the immune and stromal scores in most cancer types, which indicated that the PBK expression level is negatively correlated with tumor immune infiltration. We also found that the PBK level was significantly correlated with the degree of infiltration of M0 and M1 macrophages, T cells CD4 memory activated, and T cells follicular helper, consistent with the tumor microenvironment analysis that PBK is negatively related to immune infiltration.

Furthermore, GSEA analysis revealed that PBK participated in a wide range of functions and pathways relevant to the cell cycle and DNA replication, including mitotic nuclear division, sister chromatid segregation, the DNA integrity checkpoint, and the mitotic cell cycle checkpoint in GO terms, as well as the cell cycle and DNA replication in KEGG terms. All these terms were enriched in the PBK high-expression side, which suggested that high PBK expression mainly involved these signaling pathways, participated in mitosis and the cell cycle, and may also function in promoting tumor cell proliferation. These data are consistent with previous reports that PBK is involved in mitosis and upregulates in multiple cancers and promotes their proliferation. Kar et al. (2019) reported that PBK was overexpressed in ACC samples and correlated with poor survival, and targeting PBK in ACC cell lines decreased the function of cell proliferation, clonogenicity, and anchorage-independent growth. Diao et al. (2019) reported that the employment of a PBK/TOPK inhibitor could inhibit the proliferation of lung cancer cells. PBK also elevates in breast cancer and serves as a downstream target of Hippo-YAP signaling and promotes the proliferation of breast cancer cells by mediating the geranylgeranylation signaling pathway (Dou et al., 2015).

Recent study has suggested that overexpression of PBK decreased ovarian cancer responsiveness to cisplatin treatment through inducing autophagy *in vivo* (Ma et al., 2019). It is also reported that PBK/TOPK knockdown in colorectal carcinoma cell lines increased apoptosis and G2/M arrest in tumor cells, indicating that PBK may be a target for relevant inhibitors to sensitize tumor cells to chemotherapy-induced apoptosis (Hu et al., 2010). The IC50 value, the concentration of drug required for 50% inhibition, can be used to measure the ability of a drug to induce tumor cell apoptosis. Based on GDSC data, we performed the analysis to find out the association between PBK expression levels with IC50 values of different drugs in pan-cancer, and we found that the PBK expression level was positively correlated with trametinib, RDEA119, PD032590, and selumetinib, which indicated that PBK may be positively correlated with the treatment of these chemotherapeutic drugs and may provide a new direction in tumor chemotherapy. The effectiveness of these drugs in certain cancer types requires more comprehensive experimental and clinical studies in the future to validate it.

There are some limitations in our study. There are few patients’ data in some rare cancer types, which may lead to inaccurate or false-positive results. Also, our analysis is based on the public datasets and the lack of our own cohorts, and the predicted results still need further experimental and clinical studies to confirm them. Importantly, our study is just an investigation at the bioinformatic level without cells, animals, or patients’ samples being experimented upon. This study would provide a fundamental view of PBK in various cancers for further cancer research.

In summary, our analysis indicated that PBK was significantly upregulated in various cancers and served as a biomarker in multiple tumor progress and patient survival. Also, high PBK expression is involved in the cell cycle and relevant biological functions and signaling pathways in different cancer types. Moreover, PBK expression was strongly correlated with TMB, MSI, and certain immune checkpoints’ expression across various cancer types, while being negatively correlated with the immune infiltration. Moreover, PBK is positively correlated with the effectiveness of some chemotherapeutics such as trametinib, RDEA119, PD032590, and selumetinib. Our study may help us to understand the oncogenic role of PBK in the origination and progression of tumors, and more experiments still need to be carried out in the future.

## Data Availability

The original contributions presented in the study are included in the article/Supplementary Material; further inquiries can be directed to the corresponding author.
