# Supplementary figures and images for: An Integrative Pan-Cancer Analysis of PBK in Human Tumors
Source: Front Mol Biosci. 2021 Nov 10;8:755911. doi: 10.3389/fmolb.2021.755911 (PMC8631476; doi:10.3389/fmolb.2021.755911)

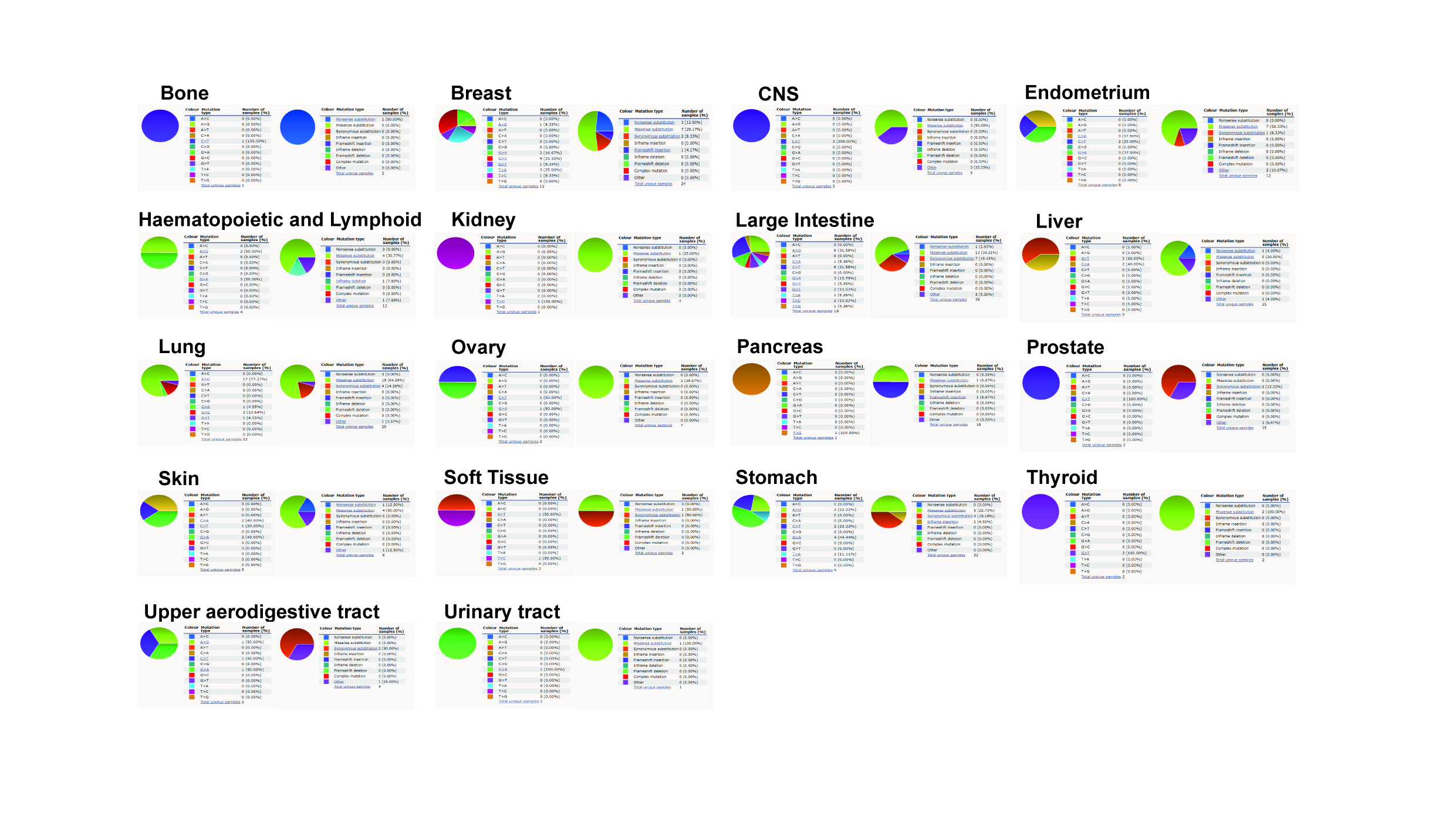

Supplement: Supplementary file 3 [file Image1.tif]
